# Misinformation About Orthopaedic Conditions on Social Media: Analysis of TikTok and Instagram

**DOI:** 10.7759/cureus.49946

**Published:** 2023-12-05

**Authors:** Oluwadamilola Kolade, Roxana Martinez, Aderemi Awe, Justin M Dubin, Nima Mehran, Mary K Mulcahey, Sean Tabaie

**Affiliations:** 1 Orthopaedics, Howard University Hospital, Washington, USA; 2 Orthopaedic Surgery, Augusta University Medical College of Georgia, Augusta, USA; 3 Urology/Andrology, Memorial Healthcare System, Miami, USA; 4 Orthopaedic Surgery, Kaiser Permanente Los Angeles Medical Center, Los Angeles, USA; 5 Orthopaedic Surgery, Loyola University Medical Center, Chicago, USA; 6 Orthopaedic Surgery, Children's National Hospital, Washington, USA

**Keywords:** social media, content quality, healthcare professionals, instagram, tik tok, orthopaedic surgery

## Abstract

Introduction

Social media outlets such as TikTok (TT) and Instagram (IG) have surged as a method to disseminate information. More recently, healthcare professionals have targeted this space as a means to provide medical education and advice. With the ever-growing content on these applications, there is significant variability and quality of material available, which can lead to the dissemination of misinformation. This study aims to evaluate the accuracy and popularity of content on common orthopaedic pathology on TT and IG.

Methods

Content on TT and IG related to six common orthopaedic conditions - achilles tendon tear, ACL tear, meniscus tear, tennis elbow, rotator cuff tear, and ankle sprains - was evaluated between April and June 2022. The top ten posts for the top two associated hashtags for each condition were reviewed. The quality of each post was analyzed using the DISCERN instrument, rating each on a scale of 1 to 5. Each post was characterized by the author's profession (physician, physical therapist, chiropractor, etc.) and content type (educational, testimonial, personal, promotional, and entertainment). Popularity and engagement metrics such as “comments,” “likes,” and “shares” were also collected.

Results

There were 165,666,490 views on TT and 9,631,015 views on IG amongst the six common aforementioned orthopaedic conditions. Content created by physicians had less overall engagement (16.1%) compared to content created by non-physicians (83.9%). The quality of content on average was low (mean misinformation index 2.04 ± 1.08 (1-5)^1^. Physician-created posts in comparison to non-physician posts were significantly more accurate (mean misinformation index score 3.38 ± 1.12 vs 1.89 ± 0.94, p<0.0001).

Conclusions

Common orthopaedic conditions such as Achilles tendon tears, ACL tears, and meniscus tears are frequently the focus of content posted on TT and IG; however, this information is often not medically accurate. Increased physician engagement may help to rectify this misinformation

## Introduction

The rising role of social media (SoMe) in disseminating medical information cannot be understated. The term “social media” refers to Internet tools that allow individuals and communities to gather and share information, opinions, photos, videos, and other content within Internet applications [1,2]. In the United States, 244 million people or about 80% of the population, use the Internet for social networking [3]. Instagram (IG) and TikTok (TT) are both popular SoMe platforms, the former being a platform where images, short videos, and posts are shared, while the latter is exclusively a short-video-based platform. Both platforms have over 1 billion users per month. The COVID-19 pandemic catalyzed the growth of all SoMe platforms, growing the number of Instagram and TikTok users by 49.5% and 85.3%, respectively, in 2020 [4].

Given SoMe’s ease of use and continued growth, it has quickly become an easy, accessible source of medical information for patients. In 2021, nearly three-quarters of internet users claimed to use the internet to look up health information [5]. For example, COVID-19-related videos on TikTok were viewed approximately 93.1 billion times. Ramkumar et al. found that more than 40% of healthcare consumers utilized social media for their healthcare information needs, with an even larger percentage of consumers in the younger age groups [6]. For consumers aged 18-24, 90% believed information presented on social media to be completely accurate [7].

SoMe, however, has the potential drawback of disseminating medical misinformation. Other surgical subspecialties have investigated this. Steimle et al., for instance, found that the majority of popular urologic posts on IG and TT were not considered medically accurate and that only a minority of posts were created by physicians [3]. Previous orthopaedic studies evaluating content on clubfoot and carpal tunnels on YouTube and popular search engines (e.g., Google) have also been found to be inaccurate [8,9]. This echoes the results of older studies [9]. Currently, the extent and quality of common orthopaedic conditions on SoMe - especially on fast-growing platforms like TT and IG - remain unknown.

In light of this information, the purpose of this study was twofold: to quantify the amount and popularity of content related to common orthopaedic conditions on IG and TT and to grade the accuracy of top posts. We hypothesized that common orthopaedic conditions are popular on these social media platforms and that the quality and accuracy of posts are poor.

## Materials and methods

Selection of orthopaedic topics

As there are a wide range of orthopaedic conditions, we decided to narrow our search to comprise the topics that appear to be of the greatest interest to the general community. A literature search was performed to identify the most frequently diagnosed orthopaedic conditions as per the Centres of Medicaid and Medicare Services and the Agency for Healthcare Research and Quality [10]. This was identified by the frequency of ICD-10 codes utilized. These two approaches were used in parallel, and the results were compared. The results were generally concordant; therefore, no further selection or arbitration process was deemed necessary.

Evaluation of topic interest

This study involved the assessment of publicly available social media posts and, as a result, was considered “IRB exempt.” All posts were independently examined by two authors (A1 and A2). The determination of interest in each topic was quantified by searching each topic in each application. On TT, the search bar function allows users to input the topic of interest and then select correlated words or phrases preceded by the pound (#) symbol. These labels (e.g., “ACL tear”) can then be selected, directing the user to the top posts with the associated label. We included the two most popular labels (colloquially known as a “hashtag”) for each orthopaedic condition. This resulted in 12 separate hashtags: #achillestendon, #achillestendonitis, #acltear, #aclrecovery, #rotatorcuff, #rotatorcuffsurgery, #meniscus, #meniscustear, #tenniselbow, #tenniselbowrelief, #anklesprain, and #anklesprainrehab. The same search was done on IG, resulting in 12 separate hashtags: #achillestendon, #achillestendonitis, #acltear, #aclrecovery, #rotatorcuff, #rotatorcuffsurgery, #meniscus, #meniscustear, #tenniselbow, #tenniselbowtreatment, #anklesprain, and #anklesprainrehab. Data collection occurred between April 2022 and June 2022. Posts were excluded if they were in a language other than English.

Characterization and accuracy of data

Data collection guidelines were formulated prior to the initiation of the study to standardize data collection and minimize variability between investigators.

The author of the post was characterized as either a physician or a non-physician (e.g., patient, company, academic institution, physical therapist). The categorization of the author was determined by evaluating the social media profile of the person or group posting. Any confusion about the author’s credentials was resolved by viewing other posts and performing a Google search to ascertain the author's occupation and if the author was a physician. Each post was then assigned a topic category: educational, promotional, testimonial, personal, and entertainment. For instance, a post committed to promoting a product, company, or service was deemed promotional. This would differ from a post that was purely educational and created by a physician or healthcare worker to educate the social media audience. Both reviewing authors agreed on each post categorization.

Both IG and TT use a complex algorithm that takes into account the number of likes, shares, views, and comments to increase the visibility of more popular content. Due to this, the number of likes, shares, and comments was deemed an appropriate index of popularity. These metrics were collected and used as a barometer of popularity and viewer engagement for each post.

**Figure 1:**
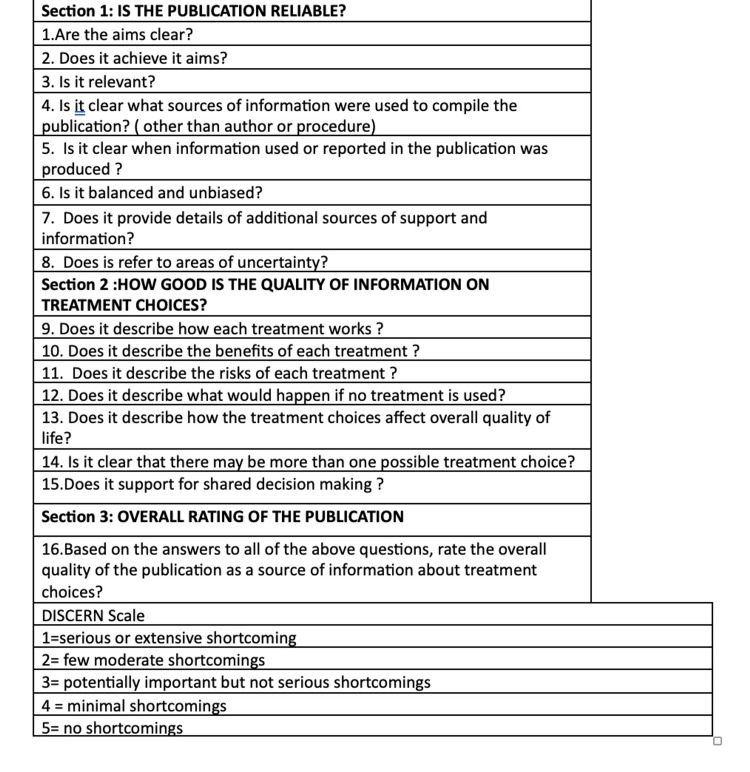
Discern Instrument Image Credits: Oluwadamilola Kolade, Author

Each post was independently examined by two authors (A1 and A2). Content analysis was performed using the DISCERN instrument to examine the quality of information on either IG or TT [7,11]. The DISCERN instrument is a validated tool to evaluate the quality of written consumer health information [11,12]. This instrument consists of 16 questions, divided into three sections. Section 1 focuses on the reliability of the information; section 2 addresses the specific aspects of the treatment, including risks, benefits, and alternatives; and finally, the third section asks about the overall impression of the content. Each post was evaluated for accuracy and scored for the degree of misinformation on a scale of 1 to 5, where a score of 5 indicates the highest accuracy and a score of 1 indicates the lowest accuracy (Figure 1).

Statistics

Descriptive statistics were reported. A two-sided T-test was used to compare the averages of continuous variables. Mean scores are reported in the text, with standard deviations (SDs) enclosed in parentheses. The statistical analysis was performed using R Studio (R Studio; Boston, MA, USA). Statistical significance was considered at p<0.05.

## Results

Content reach and engagement

A total of 120 posts were analyzed. Overall, TT had 165,666,490 views, and IG had 9,631,015 views across the six common orthopaedic conditions. Among all topics, posts related to Achilles tendon tears had the most views on TT (37,611,655; 22.7%), followed by ACL tears (36,205,855; 21.9%) and rotator cuff tears (26,790,912; 16.2%). On IG, posts related to rotator cuff tears had the most views (5,029,521, 52.2%), followed by Achilles tendon tears (2,379,260, 24.7%) and ACL tears (1,213,589, 12.6%) (Tables 1-2).

**Table 1:** Tiktok views for each orthopaedic pathology

Topic	Tik-Tok views	Percent %
Achilles tendon	37,611,655	22.7%
ACL tear	36,205,855	21.9%
Rotator cuff	26,790,912	16.2%
Meniscal tear	24,393,327	14.7%
Tennis elbow	20,346,270	12.9%
Ankle sprain	20,318,471	12.3%
Total	165,666,490	100%

**Table 2:** Instagram views for each orthopaedic pathology

Topic	IG views	Percent %
Rotator cuff	5,029,521	52.2%
Achilles tendon	2,379,260	24.7%
ACL tear	1,213,589	12.6%
Meniscal tear	750,325	7.79%
Tennis elbow	159,914	1.66%
Ankle sprain	98,406	1.02%
Total	9,631,015	100%

Sampled posts on TT received a total of 4,281,791 likes, 23,906 comments, and 88,477 shares (Tables 3-5). Posts related to ACL tears had a high level of engagement, comprising 42.9% (n = 1,837,171) of all likes and 42.7% (n = 10,206) of all comments. This was followed by posts related to rotator cuff tears, compromising 1,400,988 (32.7%) of TT likes and 4,490 (18.8%) of TT comments. Thirty-eight percent of shared posts were about rotator cuff pathology, followed by 23% of shared posts related to ACL tears. TT accounts with posts related to tennis elbow had the most followers, accounting for 15,82,667 followers (46.9%) (Table 6).

**Table 3:** TikTok likes physicians versus non-physicians

TT content likes	Total	%	Physicians	%	Non-physicians	%
ACL tear	1,837,171	42.91	501,752	96.26	1,335,419	35.51
Rotator cuff	1,400,988	32.72	6994	1.34	1,393,994	37.07
Ankle sprain	406,052	9.48	5137	0.99	400,915	10.66
Meniscal tear	288,902	6.75	4748	0.91	284,154	7.56
Achilles tendon	222,322	5.19	2597	0.50	219,725	5.84
Tennis elbow	126,356	2.95	0	0.00	126,356	3.36
Total	4,281,791	100.00	521,228	100.00	3,760,563	100.00

**Table 4:** TikTok comments physicians versus non-physicians

TT content comment	Total	%	Physician comments	%	Non-physicians	%
ACL tear	10,206	42.69	6957	88.31	3249	20.27
Rotator cuff	4490	18.78	432	5.48	4058	25.32
Ankle sprain	2367	9.90	111	1.41	2256	14.08
Meniscal tear	2037	8.52	330	4.19	1707	10.65
Tennis elbow	1509	6.31	0	0.00	1509	9.41
Total	23,906	100	7878	100	16,028	100.00

**Table 5:** TikTok shares physicians versus non-physicians

TT content shares	Total	%	Physicians	%	Non-physicians	%
Rotator cuff	34,349	38.82	858	4.49	33,491	48.29
ACL tear	20,782	23.49	17,532	91.66	3250	4.69
Achilles tendon	12,659	14.31	240	1.25	12,419	17.91
Tennis elbow	8153	9.21	0	0.00	8153	11.76
Ankle sprain	8627	9.75	62	0.32	8565	12.35
Meniscal tear	3907	4.42	436	2.28	3471	5.01
Total	88,477	100.00	19,128	100.00	69,349	100.00

**Table 6:** TikTok and Instagram account followers comparison of physicians and non-physicians

Account followers per topic	TT Total	%	MD total	Non-MD total	IG Total	%
Achilles tendon	4,840,129	14.15	226,200	4,613,929	2,386,543	7.63
ACL tear	2,147,000	6.28	2,027,900	119,100	3,697,886	11.83
Rotator cuff	3,049,747	8.92	326,896	2,722,851	7,431,800	23.78
Meniscal tear	2,273,115	6.65	968,100	1,305,015	5,975,253	19.12
Tennis elbow	15,862,667	46.39	0	15,862,667	4,838,411	15.48
Ankle sprain	6,021,511	17.61	1,500,000	4,521,511	6,928,322	22.16
Total	34,194,169	100%	5,049,096	29,145,073	31,258,215	100.00

Sampled posts on Instagram received a total of 178,896 likes, 208,794 comments, and 48,777 shares (Table 7). The topic of rotator cuff tears had a high level of engagement, comprising the majority of comments and shares (37.1% and 49.5%, respectively) and 18.4% of likes (Table 8). Tennis elbow had the most likes (n = 61,542 likes), accounting for 34.40% of likes. Authors with top posts for rotator cuff tears had the most followers on IG, accounting for 23.8% of followers (n = 7,431,800) (Table 6).

**Table 7:** Instagram likes for each orthopaedic pathology

IG topic	Likes	%
Tennis elbow	61,542	34.40
Rotator cuff	32,976	18.43
Ankle sprain	31,893	17.83
ACL tear	23,371	13.06
Achilles tendon	18,218	10.18
Meniscal tear	10,896	6.09
Total	178,896	100

**Table 8:** Instagram shares for each orthopaedic pathology

IG Topic	Shares	%
Rotator cuff	24,139	49.49
Tennis elbow	13,549	27.78
ACL tear	7245	14.85
Achilles tendon	2638	5.41
Ankle sprain	810	1.66
Meniscal tear	396	0.81
Total	48,777	100

General characteristics

The majority of posts on both TT and IG were educational in nature, accounting for 72.9% (22,787,238) and 71.9% (24,585,607) of total content, respectively (Table 9). Most posts on TT focused on Achilles tendon pathology, accounting for 22.7% (37,611,655) of views, whereas most posts on IG focused on rotator cuff tears (5,029,521, or 52.2% of all views) (Table 1). Physicians created the minority of posts on both IG and TT, accounting for 0.83% (259,443) and 11.7% (4,000,717) of posts, respectively. Posts created by physicians accounted for the lowest number of TT likes (12.2%; n = 521,228), comments (33%; n = 7,878), and shares (21.6%; n = 19,128) (Tables 7, 8, 10). The same was found for IG posts created by physicians, accounting for <1% of likes (1484) and comments (1732).

**Table 9:** Content categories for Instagram and TikTok posts

Content category N%	Instagram	Tik Tok
Educational	72.9%	71.9%
Promotional	17.8%	5.6%
Testimonial	4.67%	0
Personal	2.8%	22.4%
Entertainment	1.86%	4.67%
Physician posts	0.83%	11.7%
Non-physicians posts	99.1%	88.2%

**Table 10:** Instagram comments for each orthopaedic pathology

IG topic	Comments	%
Rotator cuff	77,528	37.13
Ankle sprain	72,395	34.67
ACL tear	19,091	9.14
Meniscal tear	16,211	7.76
Tennis elbow	12,727	6.10
Achilles tendon	10,842	5.19
Total	208,794	100

Of the TT posts created by physicians, content related to ACL tears accounted for the majority of likes (n = 1,837,171; 96.3%), comments (n = 10,206; 88.3%), and shares (n = 20,782, 91.2%). Of the TT posts created by non-physicians, content related to rotator cuff tears accounted for the most likes (n = 1393994, 37.1%) and comments (n = 4058, 25.3%). Since there was a paucity of physician-created content in the IG group, a subdivision analysis was not performed.

Content accuracy

Across all orthopaedic conditions evaluated on TT, educational posts had a mean misinformation index score of 2.04 ± 1.08 on scales 1-5 (Table 11). Among IG educational posts, there was a mean misinformation index of 2.11. Among TT posts, physician posts were significantly more accurate than non-physician posts (3.38 ± 1.12 vs. 1.89 ± 0.94, p-value = 0.00002). The most accurate topic discussed on TT was meniscal tear, with an average score of 3 on a scale of 1-5. The least accurate topic on TT was ACL tear, with an average score of 1.28 ± 0.73 on a scale of 1-5. Of note, tennis elbow was the only topic that did not have at least one physician post. Physician posts were significantly more accurate for the topics of ACL tears, rotator cuff tears, and meniscus tears. There was no statistically significant difference found for the topics of Achilles tendon tears or ankle sprains. 

**Table 11:** TikTok and Instagram misinformation indices physicians compared to non-physicians post *No physician data points **One physician data point ***As there were limited posts by physicians in this group, subdivision analysis was not performed

TT misinformation indices	Overall (SD)	Physicians (SD)	Non-physicians (SD)	P-value	IG misinformation indices***
Achilles tendon	2 (0.94)	3 (1.41)	1.875 (0.82)	0.089	2
ACL tear	1.28 (0.73)	2 (1)	1 (.25)	3.201 × 10^−^^5^	1.81
Rotator cuff	2.10 (1.06)	3.66 (0.76)	1.911 (0.98)	0.013	2.23
Meniscal tear	2.25 (1.46)	4.5 (0.29)	1.88 (1.24)	0.0018	2
Tennis elbow	2.125 (0.81)	0 (0)*	2.19 (0.96)	0.038	2.23
Ankle sprain	2.025 (0.80)	3 (0)**	2.02 (0.69)	0.26	2.38
Total	2.04 (1.08)	3.38 (1.12)	1.89 (0.94)	2.756 × 10^−^^5^	2.11

## Discussion

With the popularity and ease of access to social media, many people use it as a method of acquiring medical information [7,13,14]. This is the first study to evaluate the quantity and quality of information on common orthopaedic conditions on two of the most popular SoMe platforms, TT and IG.

Our study found that common orthopaedic conditions are frequently discussed on SoMe applications, with our sampled cohort having over 16 million views on TT and 9 million views on IG. Despite the vast amount of content on orthopaedic conditions in SoMe, our study finds that the quality of the information is low. These data are consistent with previous studies examining orthopaedic content. Jang et al. evaluated TT content on scoliosis, concluding that videos on average had poor quality as per DISCERN and Scoliosis Exercise Education scores and that a lower DISCERN score correlated with the most user engagement [15]. Their study did not distinguish whether posts were made by physicians. Our study, however, found that approximately 90% and 99% of posts were from non-physicians on TT and IG, respectively, indicating a lack of not only an orthopaedic surgeon but physician involvement in general when it came to content creation. Interestingly, our study found that non-physicians accounts had approximately 6 times the number of followers compared to physicians' accounts (30 million versus 6 million). This disparity in followers underscores both the impressive reach of non-physician content creators as well as the variability in the information provided on these platforms. Although the content created by non-physician creators does add to the discussion on common orthopaedic content, our study demonstrates that there is a higher probability of the content being less factual. It additionally raises the question: why do physicians have fewer followers and apparent reach when compared to non-physicians? This under-utilization of social media by physicians has been found in several sub-specialties within orthopaedics [2,4,7,16]. This exemplifies the need for a multidisciplinary approach to creating posts that are both accurate and attractive to the public, involving a collaborative effort between professional content creators and orthopaedic surgeons. As social media platforms become more prominent in our daily practice of medicine, it is crucial we understand how these applications can be better used to guide the decision-making process between doctors and patients [7].

In this study, we found that physician content had statistically higher misinformation indices when compared to non-physician content, suggesting that generally, there is higher accuracy among physician posts. These results held true with the most engaging topics - ACL and rotator cuff tears - where most of the posts were made by non-physicians. Ramkumar et al. [9] performed a retrospective analysis of 3145 ACL-related posts on IG and Twitter, finding that 92% of posts were made by patients [7,17]. They additionally found that across 97 National Football League (NFL) surgeon team surgeons, none had Instagram accounts, and only 16% had Twitter accounts [5]. In a separate study evaluating shoulder and elbow surgery posts across IG and Twitter, Ramkumar et al. found that only 37% of surgeons from the top 5 ranked U.S. News and World Report institutions had active accounts; of the physician-made posts in the study, 87% were advertisements for their practice [18]. The well-documented absence of physician-created, education-based posts for these popular orthopaedic topics, combined with our findings, suggests a potential area for intervention by orthopaedic surgeons to improve content quality. This effort can additionally be addressed by national organizations such as the Academy of American Orthopaedic Surgeons and the American Orthopaedic Organization. In addition to creating their own content, they may help navigate the field entirely by introducing guidelines for SoMe orthopaedic-related content. Orthopaedic surgeons and these national organizations can work in conjunction to improve content quality and prevent abuse from private companies motivated by financial gain.

There are some limitations to our study that should be acknowledged. The majority of post-assessments were performed by two co-authors, with a third consulted, if necessary, to help resolve any discrepancies. As the reviewers were orthopaedic surgery residents, there was a potential for subjective potential bias. There was selection bias in the posts evaluated, as only those with the top two hashtags for the reach topic were included in this sample. In addition, this study was a cross-sectional study dependent on the most popular posts/videos over a two-month period. Since the popularity of both IG and TT posts is dynamic and time-sensitive, the validity of the engagement of a post has the potential to vary over time. There are no well-established tools to grade the quality or accuracy of health-based information that can be translated across all media forms. An additional limitation is that our study concentrated solely on IG and TT, excluding other applications such as Twitter and Facebook, both of which can impact the perceptions society has of common orthopaedic conditions. Finally, our study did not include content from private accounts, prohibiting the assessment of both their posts and interactions with SoMe content.

## Conclusions

We conducted the first study to analyze the quality of orthopaedic content on Instagram and TikTok. Our findings illuminate the overall low calibre of information on these platforms while also demonstrating that most of the content did not come from physicians. It also highlights that physician-made posts are more reliable and accurate than those by non-physicians. As experts in the field, we have to disseminate the appropriate information to educate patients on orthopaedic conditions while also managing patient expectations. There is a role for physician-created content to display a clear narrative of preoperative care, an explanation of indications for both operative and nonoperative management, intraoperative treatment, and postoperative regiments. Recognizing the possible benefits of SoMe engagement as well as the limited involvement with SoMe, we encourage the increased social media presence of both orthopaedic surgeons and associations to better educate and empower patients.
